# Immune Checkpoint Inhibition in Classical Hodgkin Lymphoma: From Early Achievements towards New Perspectives

**DOI:** 10.1155/2019/9513701

**Published:** 2019-05-07

**Authors:** Diego De Goycoechea, Gregoire Stalder, Filipe Martins, Michel A. Duchosal

**Affiliations:** ^1^Service and Central Laboratory of Hematology, Centre Hospitalier Universitaire Vaudois and University of Lausanne, rue du Bugnon 46, CH-1011 Lausanne, Switzerland; ^2^School of Life Sciences, Ecole Polytechnique Fédérale de Lausanne (EPFL), 1015 Lausanne, Switzerland

## Abstract

Immune checkpoint inhibition (ICI) became one of the major breakthroughs in cancer treatment over the past decade and entered into therapy within standard oncohematology practice. ICI has demonstrated impressive response rates as salvage therapy in relapsed/refractory (R/R) classical Hodgkin lymphoma (cHL) and is now being tested as an adjunction to chemotherapy in the frontline settings. CHL exquisite sensitivity to PD-1/PD-L1 axis inhibition relies on a particular biological background. By contrast, non-Hodgkin lymphomas (NHL) have demonstrated heterogeneous response rates using ICI. These observations highlight discrepancies between various types of lymphomas in terms of genetic alterations, immune microenvironment interactions, and disease phenotype. This review aims to focus on cHL immune escape mechanisms, focusing on cHL biological sensitivity to PD-1 blockade. We will summarize the available data issued from clinical trials on ICI in cHL and its safety profile. Going beyond the current use of monoclonal antibodies (mAb) targeting immune checkpoints in clinical practice, we will offer an overview of new combinatory therapeutic perspectives where cHL immunotherapy may be considered.

## 1. Introduction

Accounting for a tenth of lymphoma cases, classical Hodgkin lymphoma (cHL) is characterized by peculiar histologic and immunologic features [1]. A striking inflammatory infiltrate surrounding rare multinucleated giant cells were originally reported by Dorothy Reed more than one hundred years ago. This observation already highlighted the intriguing immune repercussion of cHL where authors noticed tuberculin anergy in affected patients [2]. CHL-associated cellular immunosuppression, which translates into an increased infectious risk that may precede disease by several years, was also further supported by the necessity of delivering irradiated blood products to avoid the risk of transfusion-associated graft-versus host disease (GVHD) in these patients [3]. Although considered a curable disease in almost 80% of cases, relapse cases of cHL are still challenging. Rescue and intensive chemotherapies followed by autologous hematopoietic stem cell transplantation (auto-HSCT) can put into remission about half of the patients [4].

The subset of patients necessitating further treatment in the cases of second relapse or refractory disease is considered for allogeneic HSCT (allo-HSCT). In this situation, a survival plateau has been difficult to reach at least until recently [5].

The impressive results of nivolumab (a fully human IgG4 monoclonal antibody against PD-1) in R/R cHL led to its FDA approval in 2016 [6, 7]. Demonstrating an objective response rate (ORR) of 66.3% in the Checkmate 205 trial, nivolumab's unprecedented performance made it a robust alternative bridge therapy to allo-HSCT [8]. CHL exquisite sensitivity to PD-1 blockade relies on lymphoma cell genetic alterations and particular tumor microenvironment (TME) inflammatory phenotype. In an attempt to optimize the first-line treatment of cHL, PD-1 blockade is now being tested as an adjunct to doxorubicin, vinblastine, and dacarbazine (“AVD” regimen) in Europe and USA in both early and advanced stages (NCT03004833 and NCT03033914 trials), respectively. Preliminary results have shown high response rates with an acceptable safety profile in the frontline setting with patients achieving complete responses (CR) in 67-80% of cases [9, 10]. High expectations regarding PD-1 blockade in cHL reside in its potential to decrease treatment-related toxicity of current intensive chemotherapy regimen, such as bleomycin-related pulmonary toxicity, and to challenge the place of adjuvant radiotherapy in affected young populations [11]. Avoiding bleomycin may reduce the rate of fatal pulmonary toxicities, which has been reported in 4-5% of cHL patients in a systematic review [11]. Anti-PD-1 mAb are also being studied as an upfront treatment in patients unsuitable for standard therapy (NCT03331731).

Results of PD-1 blockade efficacy in R/R NHL are more variable: it is effective to treat primary mediastinal B-cell lymphoma (PMBCL) [12], Grey-Zone lymphoma [13], CNS primary diffuse large B-cell lymphoma (CNS-DLBCL), and primary testicular lymphoma (PTL) [14], where PD-1 inhibition significantly affects response rates. Heterogeneous immune escape pathways' expression and variable immunosuppressive properties of NHL explain in part these disparities [15].

Focusing on cHL as a paradigm for its high sensitivity to ICI, this review brings insights into the biological background behind its effectiveness. It also reflects on ICI current place in patient care and provides an overview of the strategies being foreseen to boost its effects in the future.

## 2. cHL Microenvironment and Immune Escape Mechanisms

CHL is a malignancy issued from giant, often multinucleated cells, called Reed-Sternberg (HRS). These cells constitute less than 5% of the tumor bulk, and they grow and survive with the help of interactions with and within a heterogeneous background of inflammatory cells. Germinal center B-cells are considered to be the origin of HRS cells [16].

In the era of polychemotherapy and radiation therapy, the various subtypes of cHL, namely, nodular sclerosis, lymphocyte-rich, lymphocyte-depleted, and mixed cellularity subtypes, demonstrating the heterogeneity of their underlying biology, do not translate into direct consequences for patient care [17]. The latter is mainly driven by disease stage and other risk features [18, 19]. However, the underlying biology of cHL offers now new prognostic markers and may regain the interest of clinicians in this new era of immunotherapy. Collectively, these histological subtypes share a variable number of infiltrating lymphocytes, generally forming the main component of the tumor cell environment, monocyte-macrophages, eosinophils, neutrophils, plasma cells, and mesenchymal stromal cells (MSC), such as endothelial cells and fibroblasts associated with a variable degree of tissue extracellular matrix. These infiltrative components are necessary to promote HRS growth. This has been demonstrated by HRS absence of growth in* ex vivo* experiments and indirectly by substantial difficulties to establish cell lines where interstitial cells are lacking [20]. Reciprocally, HRS cells shape their microenvironment to benefit in return from growth and survival signals provided by surrounding inflammatory cells. Directly and indirectly, HRS attract surrounding cells via chemokine secretion. This is valid for neutrophils (IL-8), eosinophils (CCL5, eotaxin), macrophages (CCL5), mast cells (CCL5), T regulatory lymphocytes (CCL5, CCL17, CCL20, CCL22), and type 2 helper T-cells (CCL17, CCL22). These environmental cells provide not only survival/support signals for HRS, but also, for part of them, immune escape signals [21–24].

Main HRS survival signals lead to NF-kB pathway hyperactivation [25]. They originate from CD40, CD30, TACI, and BCMA receptors engagement by their respective ligands located on the surface of surrounding CD4+ T-cells, eosinophils and mast cells (i.e. CD40L and CD30L), and/or secreted molecules from myeloid-derived cells and neutrophils for BAFF and APRIL, respectively [26, 27].

**Figure 1:**
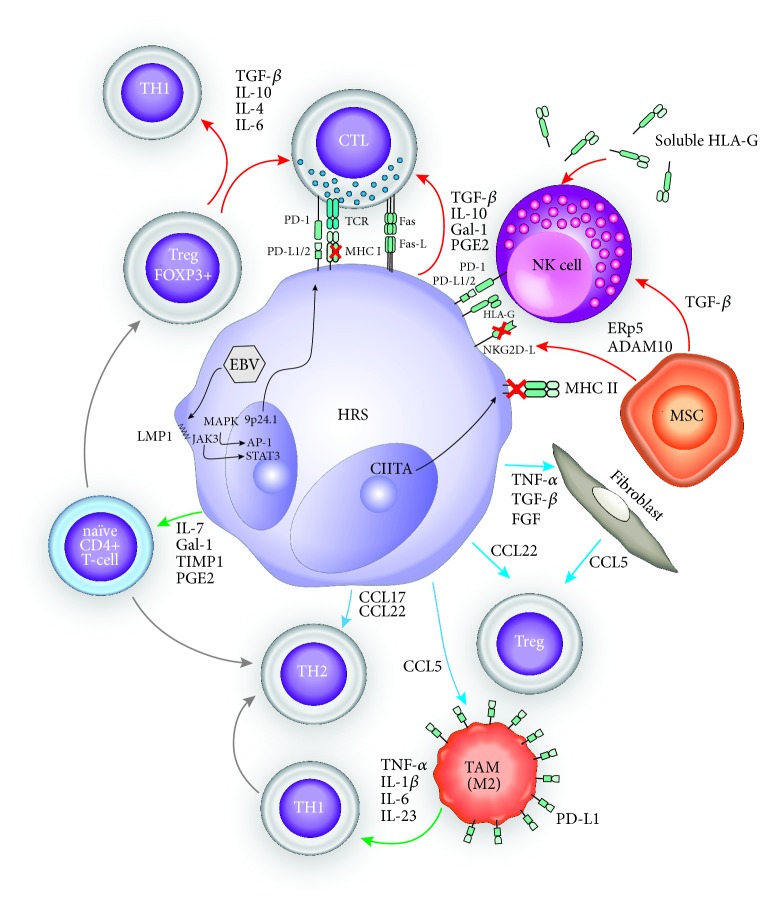
*Immune-escape mechanisms of cHL TME*. Chemokine secretion by HRS plays a central role in the TME immunosuppressive state of cHL. They allow differentiation of infiltrating naïve CD4+ T-cells into regulatory FOXP3+ and Th2 T-cells and provide CTL inhibitory signals through TGF-ß, IL-10, galectin-1 (Gal-1), tissue inhibitor of metalloproteinase 1 (TIMP1), and prostaglandin E2 (PGE2). HRS also attract Tregs/Th2 T-cells from the systemic circulation, through the secretion of CCL22 and CCL17, respectively, while at the same time promoting their expansion through the secretion of Gal-1, TIMP1, and PGE2. Fibroblasts also contribute to Treg chemoattraction through CCL5 secretion. In the same line, TAM promote the differentiation of Th1 cells towards the Th2 phenotype. Nevertheless, recent evidence challenges this concept of predominant Th2 polarized TME and evidenced an increase in activated Th1 T-cells in TME of cHL patients. EBV latent infection also plays a key role, through the production of LMP1 which activates the MAPK and JAK/STAT3 pathways leading to transcriptional activation of the 9p24.1 locus with consequent PD-L1/2 overexpression [5]. Even in EBV negative cHL, PD-L1 (and, to a lesser degree, PD-L2) expression at the surface of HRS cells is still high. PD-1-PD-L1 interaction triggers the inhibition of CTL function. TAM display a high surface PD-L1 expression, thus promoting PD-1-PD-L1 axis immune escape. EBV- cHL displays also a decrease in MHC class I expression, in comparison to their EBV positive counterparts mainly through B2-microglobulin subunit downregulation. MHC class II expression can also be impaired through epigenetic silencing, in the subset of mutated/translocated CIITA cHL. In this context, it is presumed that the PD-1/PD-L1/2 axis mediates immune escape, in first instance, by dampening NK cell activity. NK function is further downregulated by TGF-ß secreted by HRS and mesenchymal MSC. MSC also edit the surface expression of NKG2D-L through the enzymatic activity of secreted ERp5 and ADAM10. HRS cells also express Fas ligand at their surface, thus promoting apoptosis of interacting Th1 and cytotoxic T-cells [88]. Red arrows: “inhibition signal”; green arrows: “differentiation signal”; blue arrows: “chemoattraction” signal.

To counteract immune tumor rejection, HRS shape the TME to induce immune tolerance (Figure 1). HRS cells are coated with a large variety of immune checkpoint ligands and transmembrane receptors mediating exhaustion of infiltrating cytotoxic and type 1 helper T-cell subsets. Cytotoxic T-lymphocyte antigen 4 (CTLA-4), membrane-bound TGF-*β*, and PD-L1 (B7-H1) expression are some of the main Treg contact mediators acting in this exhaustion process. Among these, PD-1/PD-L1 axis constitutes one of the major escape mechanisms in cHL, as demonstrated by its dense coating on HRS cells [28], which is linked to a high prevalence of 9p24.1 chromosomic amplification, a loci bearing PD-L1/2 genes [29]. A similar amplification is also frequent in some groups of NHL such as PMBCL, CNS-DLBCL, and PTL cases, thus explaining in part their higher relative sensitivity towards ICI when compared to other B-cell NHL. Chromosomal 9p24.1 copy number gains and gene amplifications also affect nearby JAK2 locus, which further intensifies PD-L1 overexpression through JAK/STAT signal pathway activation. Latent membrane protein 1 (LMP1) encoded by the inserted EBV genome mimics CD40 signaling and therefore amplifies PD-L1/L2 overexpression, through AP-1 and JAK/STAT3-mediated epigenetic control. This observation sustains a reciprocal positive biological feedback between avoidance of viral clearance and immune escape of HRS cells [30–32]. PD-1-PD-L1/2 ligation triggers T-cell phosphatase activation and consequent dephosphorylation cascade of several proteins implicated in T-cell receptor (TCR), and PI3K-AKT-to-NF-kB signaling pathways. Tyrosine phosphatases are actually recognized as important immune checkpoint modulators, and active research on their potential inhibition to boost adoptive T-cell therapy is ongoing [33]. Consequently, IL-2 and IFN*γ* secretion is also repressed, thus inhibiting T-cell cytolytic activity and cell proliferation. Stanford's pathology department recently published their evaluation of PD-L1/-L2 expression on 702 immunostained B-/T-lymphoma samples [28]. This study confirmed the high prevalence of PD-L1 positivity in cHL samples (over 80% in cHL and 75% in nodular lymphocyte-predominant HL (NLPHL), resp.). In this series, all except one PD-L1 positive cHL sample (40 over 41) were also Epstein-Barr (EBV) positive by EBER in situ hybridization, in opposition to three over the nine PD-L1-negative cHL samples.

CTLA-4 is another immune checkpoint located on the surface of T-cells which dampers the priming (early) phase of immune response. This function makes CTLA-4 a natural synergistic partner of PD-1/PD-L1 inhibition. Indeed, in addition to impeding CD28 costimulatory signaling in barring T-cells, by competing for its ligands (CD80/CD86), CTLA-4 interaction impacts also the NF-kB pathway leading to IL-2 production impairment [34, 35]. Its constitutive presence on Tregs also offers the possibility to target directly a main actor of TME immunosuppression [36]. Recent success in the treatment of melanoma, clear-cell renal cancer, and non-small cell lung cancer brings high expectations regarding ongoing trials combining both PD-1-PD-L1 and CTLA-4 inhibition in relapsing cHL (Table 1) [37–39].

Immunochemistry studies showed a high prevalence (>90%) of additional immune checkpoint regulators such as lymphocyte activation gene-3 (LAG3), T cell immunoglobulin, and mucin domain-containing protein-3 (TIM3) expressions. Expressions were found mainly on T-cells composing the TME of cHL. This was in opposition to PD-L1 displaying almost universal positivity on HRS cells. TIM3 was present in only one-third of samples included in a study assessing samples from 25 cases of cHL, while HRS were weakly LAG3-positive in a single case [40]. The implications of these findings are still unclear, even though they highlight a presumed significant role of these additional immune checkpoints within the TME component. LAG3 (CD223) is a cell surface receptor expressed by Tregs, activated B/T-cells, and antigen-presenting dendritic cells, which binds with high affinity to the major histocompatibility complex (MHC) class II. Functional consequences of its ligand engagement are cell context-dependent, promoting Treg function enhancement on the one hand, and suppressing effector T-cell function on the other hand [41, 42]. However, LAG3 signaling and intervening mechanisms of CD4/CD8 T-cell downmodulation are still poorly characterized [43]. TIM3 is an inhibitor receptor implicated in the exhaustion of cytotoxic and Th1 tumor infiltrating T-cells, although as for its former counterpart the underpinning mechanisms are still to be fully characterized [44, 45]. One of its ligands, Galectin-9, is a mediator Th1 cell death [46]. Blockade of these regulatory components is under active clinical research with several ongoing trials in solid and hematologic neoplasms, since they displayed synergism with PD-1 blockade in a preclinical setting (Table 1).

HRS also occults surface MHC class I and II in about 2/3 of cases, playing so, on both of the two-signal dependency of T-cell activation [47]. ß2-microglobulin transcription repression constitutes the main mechanism of MHC class I downregulation and seems inversely correlated with EBV status. MHC class II is also downregulated at a transcriptional level in the subset of Class II Transactivator Type I (CIITA) mutated and/or translocated cHL. MHC class II expression negativity is found in 15% to 40% of cases [48, 49]. Altogether, one-third of cHL display no expression of both MHC. These observations led to the assumption that PD-1 blockade efficiency in cHL is not primarily related to reinforce CTL immune rejection. Instead, a more pronounced effect on reversing Natural Killer cells (NK) inactivation is presumed, by impairing the interaction of PD-1 with PD-L1 located on the surface of HRS and tumor-associated macrophages (TAM). The latter are main providers of surface PD-L1 in the cHL TME because of its high density of expression on their surface [50].

NK cells are important mediators of antitumor surveillance. However, HRS cells are resistant to Fas receptor-mediated death and even in the absence of most MHC class I molecules are able to avoid NK cell activation. This is mediated by the expression of surface ß2-microglobulin-free HLA-G subunits [51]. HLA-G is mainly expressed in the placenta and plays a crucial role in its immunotolerance. A soluble form of the latter is also secreted by HRS cells and impairs NK cell extravasation and tissue migration. Finally, HLA-G can induce Treg differentiation. HRS cells display low levels of surface NKG2D ligand through secretion of proteolytic enzymes such as ERp5 (a disintegrin) and ADAM10 (a metalloproteinase domain-containing protein 10), also produced by MSC [52]. A soluble form of the NKG2D ligand is also presumably responsible for the internalization and subsequent downregulation of its receptor on circulating NK cells, thus inducing a systemic cellular dysfunction [53, 54]. TGF-ß secreted by MSCs further reinforces the downregulation of NKG2D receptor on the surface of NK and cytotoxic T-cells [55]. Several of the beforementioned characteristics of cHL immune escape, as, for example, a high number of infiltrating TAMs and a lack of MHC class I expression, negatively impact disease outcome [47, 56]. A summary of these immune escape mechanisms, together with treatment strategies under clinical investigation to overcome them, is provided in Table 1.

## 3. Immune Checkpoint Inhibition in cHL

### 3.1. Clinical Outcomes

The preclinical observation of PD-L1 overexpression in cHL led to the evaluation of ICI administration on disease evolution. To date, seven prospective clinical studies with ICI in cHL have been published (Table 2). In the first phase 1 study published in 2015 (Checkmate 039), 23 patients with R/R cHL were treated with nivolumab 3mg per kilogram of body weight every 2 weeks until complete response, tumor progression, or excessive toxicity [7]. Eighteen patients had relapsed after auto-HSCT and/or received brentuximab vedotin (BV) (an antibody-drug conjugate directed against CD30 and linked to microtubule-disrupting agent monomethyl auristatin E, MMAE) before relapse. High-grade adverse events (G3-4) occurred in 12 patients. Four patients had a complete response (CR) and 16 patients had a partial response (PR). The progression-free survival (PFS) at 24 weeks was 86%. Following this study, the same group of investigators performed a phase 2 study (Checkmate 205) including 243 patients. The latter was composed of three cohorts divided according to their treatment history: patients who did not receive BV (cohort A, n=63), patients treated with BV after auto-HSCT (cohort B, n=80), and patients who were treated with BV before and/or after auto-HCT (cohort C, n=100). The initial results from cohort B were published in the Lancet Oncology in 2016 [8]. Seven patients had a CR and 46 patients had a PR, with a PFS of 76.9% at 6 months. The results of the extended follow-up of the three cohorts were published in 2018 [57]. The overall response rate (ORR) was 69% (95% CI, 63% to 75%). Forty patients had a CR and the median PFS was 14.7 months. Response rates were similar across the three cohorts. The updated results of Checkmate 205 were presented in December 2018. Actualized ORR was 71% with 21% of patients achieving CR (Cohort A 32%, Cohort B 14%, Cohort C 20%) [58].

It should be emphasized that the Checkmate 205 study protocol was amended in July 2014 to allow patients to continue treatment beyond investigator-assessed progression if protocol-predefined criteria were met, including stable performance status and perceived clinical benefit. Patients treated beyond initial progression (TBP) were required to discontinue in the event of further progression (>10% further increase in tumor burden) [8]. Cohen reported on the 80 patients TBP over the 130 patients with progressive disease in the Checkmate 205 study. Amongst 67 evaluable patients TBP, 37 experienced stable or reduced target tumor burdens, despite the appearance of new lesions [59].

Finally, a small phase 2 study on nivolumab in relapsed cHL after treatment with BV on 17 patients with or without previous auto-HSCT was performed in Japan [60]. CR and PR occurred in four and nine patients, respectively, with a PFS of 60% at 6 months.

Another anti-PD-1 mAb, pembrolizumab, was studied in a phase 1 trial published in 2016 (Keynote 013) [61]. Thirty-one patients, all previously treated with BV with 22 of them having also received an auto-HSCT before relapse, were treated with pembrolizumab 10 mg per kilogram of body weight every 2 weeks. Five patients had a CR and 15 a PR, with a PFS of 69% and 46% at 24 and 52 weeks, respectively. Thereafter, a phase 2 study was conducted (Keynote 087), but with a dose of pembrolizumab of 200mg once every 3 weeks, based on its pharmacokinetic properties [62]. Patients were divided in three cohorts: those relapsing after auto-HSCT and subsequent BV (Cohort 1, n=69), those who were ineligible for auto-HSCT because of refractoriness to salvage chemotherapy and BV (Cohort 2, n=81), and those relapsing after auto-HSCT but without subsequent BV (although 41.7% received BV before transplantation) (Cohort 3, n=60). Among the 210 included patients, the ORR was 69% with 22.4% of CR. PFS at 6 months was 72.4%. There were no significant differences between the three cohorts. The updated results presented at ASH 2018 showed an ORR of 71.9%, with 27.6% of CR. Median PFS was 13.7 months. It seems also that cohort 2 (those with chemoresistant disease) had smaller ORR (66.7% [95%CI 55.3-76.8] vs. 76.8% [95%CI 65.1-86.1] in cohort 1 and 73.3% [95%CI 60.3-83.9] in cohort 3) and shorter PFS than the two other cohorts (11.1 months [95%CI 7.6-13.8] vs. 16.4 [95%CI 11.3-27.6] and 19.4 months [95%CI 10.8-22.1], resp.) [63].

Based on these studies, nivolumab and pembrolizumab received FDA [6, 64, 65] and UE [66, 67] approvals for the treatment of patients with cHL who relapsed or progressed after auto-HSCT and posttransplantation BV.

More recently, another anti-PD-1 mAb, sintilimab, was tested in a phase 2 trial (ORIENT-1) [68]. Ninety-two patients with R/R cHL were treated with sintilimab (200mg intravenous once every 3 weeks) until progression, death, unacceptable toxicity, or withdrawal of consent. ORR was 80.4%, with 34% CR and a PFS of 77.6% at 6 months.

Of note, exclusion criteria in all the abovementioned studies comprised allo-HSCT. Indeed, two murine models raised the concern of increased GVHD-related mortality due to ICI exposure after allo-HSCT [69, 70].

Recently, a retrospective study on 20 patients treated with nivolumab after allo-HSCT was published [71]. Six patients experienced acute GVHD, and 2 patients deceased. Noteworthily, all of these patients had already suffered a previous episode of acute GVHD. In this cohort, nivolumab did not induce chronic GVHD and no flare phenomena was noted in four patients with previously documented chronic GVHD. In this study, nine patients showed a CR under nivolumab and 10 had a PR, with a PFS at 12 months of 58.2%. Another retrospective study on 31 lymphoma patients (30 of them having cHL) treated with nivolumab (n=28) or pembrolizumab (n=2) for relapse after allo-HSCT found an ORR of 77% (15 CR, 8 PR), but with 8 (26%) GVHD-related deaths after anti-PD-1 therapy [72]. After initiation of anti-PD-1, 17 patients developed GVHD (6 acute, 4 overlap, and 7 chronic). Median PFS was 591 days.

The impact of anti-PD-1 treatment on the risk of subsequent GVHD when administered before allo-HSCT is also a matter of debate. In a retrospective study published in 2017, Merryman et al. described a cohort of 39 patients with lymphoma (31 patients with cHL), who received pembrolizumab or nivolumab and subsequently underwent allo-HSCT (median time of 62 days, range 7-260, between end of immunotherapy and allo-HSCT) [73]. They found a 1-year cumulative incidence of grade 3-4 acute GVHD of 23% and reported a PFS of 89%. An analysis of circulating lymphocyte subsets in 17 patients showed, in comparison to controls, decreased numbers of PD-1+ T cells and lower ratios of T-regulatory cells to conventional CD4+ and CD8+cells, suggesting a possible long-term implication of prior ICI treatment on the immune system after allo-HSCT. A recent review summarized the published data on the impact of prior or subsequent anti-PD-1 therapy on GVHD in patients with lymphoma (the majority of whom were cHL) treated with an allo-HSCT [74]. Among the 107 patients who received ICI before allo-HSCT, acute and chronic GVHD surged in 56% and 29% of patients, respectively. Mortality risk from GVHD was 11% in this study. One hundred and seventy-six patients treated with ICI after allo-HSCT were reported. The rates of acute and chronic GVHD were 14% and 5%, respectively, with a mortality risk from GVHD of 7%. In the absence of prospective data, recommendations for management of ICI before or after allo-HSCT are currently based on expert opinions [71]. These recommendations advocate empirically a 6-week interval between completion of anti-PD-1 therapy and allo-HSCT. In addition, these recommendations advise the use of reduced intensity conditioning regimen before allo-HSCT in this heavily pretreated patient population.

### 3.2. Safety Profile

Adverse events consecutive to ICI are distinctly different from the ones related to conventional chemotherapy. The blockade of the immunological checkpoints with mAb can trigger autoimmune complications that can affect any organ. These so-called immune-related adverse events (irAEs) vary in incidence and spectrum of affected organ systems depending on the agents used. The incidence of irAES of any grade is variable and can affect up to half of patients under anti-PD-1 therapy, such as those in the ORIENT-1 trial [68]. The incidence of high-grade irAEs among the 651 patients with R/R cHL included in different clinical trials is summarized in Table 3. The most frequent grade 3-4 adverse events according to common terminology (CTCAE v.4.0) were gastrointestinal under the form of enterocolitis (13%, 2%, and 5% in patients treated with nivolumab, pembrolizumab, and sintilimab, resp.), pulmonary (2.1% in total; including pneumonitis, dyspnea, and respiratory infections), and hepatic. Other reported high-grade adverse events encompassed general symptoms, such as fever and fatigue (1.2%), but also mucocutaneous (1%), cardiovascular (0.4%), endocrine (0.3%), rheumatological (0.3%), and renal and electrolyte (0.3%) disorders. These trials did not report fatal cases of irAEs and toxicities leading to treatment discontinuation were rare.

Hematologists should be aware of such possible complications in order to initiate early and adapted immunosuppressive treatments [75]. Most irAEs are reversible, except in the case of endocrine dysfunction, and are treated effectively by delaying the administration of the ICI and proper immunosuppressive treatments including corticosteroid and/or biological agents targeting key inflammatory cytokines such as interleukin-6 and tumor necrosis factor *α* [76].

### 3.3. Patients Not Responding to Anti-PD-1 Therapy

The radiological interpretation of tumor response is challenging in the context of immunotherapy. The assessment of tumor response using fluorodeoxyglucose-positron-emission tomography (FDG-PET) may lead, in some cases, to premature discontinuation of anti-PD-1 therapy, due to misleading imaging findings suggestive of disease progression. The immune activation and abundance of T-cell infiltration related to checkpoint inhibition has been linked to a phenomenon called “tumor flare” or “pseudoprogression” under the form of new lesions, or lesions increasing in size and metabolic activity [77]. A new biopsy or repeated imaging is advocated in these cases, mostly if the patient is experiencing clinical benefit from immunotherapy, before deciding to stop the treatment. Some patients showing these pseudoprogressions may experience late responses and even long-lasting clinical benefit from anti-PD-1 therapy. The Lugano Classification lymphoma response criteria have been refined in 2016 to address this specific issue [78]. They determined the imaging criteria suggestive of pseudoprogression (in the absence of clinical deterioration) and classified these scenarios as “indeterminate responses” necessitating additional tests in order to identify a true progressive disease. If such an eventuality is confirmed, the treatment of patients who do not respond to anti-PD-1 therapy is a main concern. Rossi and colleagues retrospectively described the treatment of 30 patients with cHL highly pretreated and who failed anti-PD-1 therapy. Seventeen patients were treated with chemotherapy alone (group 1) and 7 with chemotherapy and anti-PD-1 (group 2). ORR was 59% and 86%, respectively. This observation suggests that anti-PD-1 therapy could resensitize tumor cells to chemotherapy-induced death [79].

## 4. New Perspectives

Future aim for patients with Hodgkin's disease, as well as with other lymphoma types and more broadly with cancers, is to provide them with more efficient treatment where side effects remain as minor as possible and manageable. In this endeavor, ICI has opened a new way to treat patients, at the price of awakening autoimmunity.

It can be foreseen that efficacy of actual checkpoint inhibition, targeting PD-1/PD-L1 (and CTLA-4), could be ameliorated when we contemplate and consider using some of the myriad of checkpoint molecules involved in the interactions of immune cells capable of killing lymphoma cells [80]. Such analysis has inherent complexity that resides not only within the number of checkpoints implicated, but also in their cell specificity and time-dependent expression.

One could foresee that combining ICI with classical chemotherapy, especially in heavily treated patients, would reduce the number of antigen-presenting and immune effector cells implicated in antilymphoma immune response awakened by ICI, and globally weakening ICI. Actually, ICI are prescribed mostly in heavily treated patients, but implementing ICI earlier in the course of the disease could be more beneficial in terms of treatment efficacy.

The Specificity of ICI efficiency resides in large part within the mutational burden of the tumor leading to neoantigen formation and presentation to immune cells. The antitumor response could be boosted with tumor-derived vaccines where patients are simultaneously receiving ICI. This alternative has been evaluated in preclinical models with success and is evaluated nowadays in some cancers, including lymphomas.

Finally, combining ICI with active and passive immunization could be most beneficial. This can be done by immunizing patients with tumoral neoantigen, or by transferring to patients tumoral neoantigen-specific cells (modified or not) followed by ICI to boost the antilymphoma response. Such an avenue is currently evaluated using T cells specific for EBV in combination with PD-1-PD-L1 blockade in EBV^+^ lymphomas, including EBV^+^ Hodgkin's disease (NCT02973113).

Immune checkpoint inhibitors are all implicated in classical as well as antilymphoma immune responses. A global analysis of ICI distribution, both spatially and with time, could help to design ICI administration with better specificity (i.e., targeting IC preferentially expressed in lymphoma versus healthy tissues) and efficacy (time and length of treatment). Such an analysis has already been undertaken without always-clear conclusions. This is probably due to the complexity of the antilymphoma immune response parameters. This complexity is best illustrated by the observations that ICI are even dependent on gut microbiota [81, 82]. In the future, using a more complete picture of biomarkers would help.

Additionally, one could envisage using chemotherapeutic drugs with known immunomodulatory activities to enhance the efficacy of immune checkpoint inhibition, such as cyclophosphamide and anthracyclines. These chemotherapies can elicit an immunogenic cellular death (ICD) characterized by apoptotic bodies exposing calreticulin and releasing adenosine triphosphate (ATP) and high-mobility group box 1 protein (HMGB1) which act as an “eat me” signal towards adaptive immune cells [83–85]. Interestingly, hypomethylating agents have also been proposed to prime ICI. Interesting immunomodulatory properties of Bruton kinase inhibitors (BTK) such as ibrutinib have been pointed out in lymphoma and myeloma xenograft models [86]. Through the off-target inhibition of ITK (interleukin-2-inducible T-cell kinase), ibrutinib is able to polarize T-cell response towards à Th1 tumor rejection-prone phenotype. This property is also amenable to ICI treatment combinations and is now being tested in R/R cHL (NCT02940301).

Combining radiotherapy with ICI has the potential to boost specifically the antilymphoma efficiency of ICI while preserving the development of an immune response. Indeed, radiotherapy leads to lymphoma cell death, liberates immune-activating chemokines and cytokines, activates dendritic cell and antigen presentation, and induces effector cells (such as CTL and NK cells) activation and proliferation [87]. ICI would boost the efficiency of these effector cells, resulting in preferentially local antilymphoma efficacy. In the context of cHL known to be most sensitive to radiotherapy, this combination may be highly synergistic and may conduct therapeutic strategies using lower doses of irradiation than those in current protocols.

Last, but not least, the education of medical specialists will have to be adapted according to these novel combination therapies in terms of both available therapies and follow-up exams. One example of the latter is that response is classically evaluated using radiological exams such as scanner in combination with FDG-PET. Such an exam cannot differentiate between residual lymphoma cells and activated and proliferating immune cells around and within the tumor. This can lead to a false positive evaluation of patient under ICI treatment.

## 5. Conclusion

Since the advent of modern oncology in the mid-fifties, cHL was the standard-bearer of chemotherapy and radiotherapy early successes. Thanks to these achievements, cHL is nowadays considered a curable disease. However, a subset of patients still suffer from relapses, and despite being considered a disease of young adults, senior populations also display a peak of incidence. These relapsing/refractory patients pose a challenge for oncohematologists, regarding the ways to achieve high response rates and cures in the young patient population, but also to limit toxicities while assuring the best quality of care in the older one. PD-1 inhibition is revealed to be a valuable therapeutic option in these situations, with a high response rate and a reasonable toxicity profile. However, practitioners should not underestimate autoimmune toxicities and should be aware of the need to initiate immediate interventions if they occur. With immunotherapy being a new treatment modality, clinicians should be trained to recognize these adverse events. Many questions are still open, such as if ICI will indeed be able to decrease long-term toxicities of current standard treatment protocols, replace radiotherapy, or continue as salvage therapy, but also regarding the interpretation of treatment responses. In this new era of immunotherapy, Hodgkin's disease is once again colliding with the history of oncology, as a paradigm of ICI sensitivity and a model for therapy development.

## Figures and Tables

**Table 1:** Summary of immune escape mechanisms in cHL and alternative therapeutic strategies in development.

**Immune escape mechanisms in cHL**	**Therapeutic agents with immunomodulatory properties tested in recruiting/active clinical trials in R/R cHL**
Downregulation of MHC class I and II expression	Epigenetic modifiers in combination with immune checkpoint inhibitors∗: Decitabine + anti-PD-1 mAbs (NCT03250962)

Surface PD-L1/2 overexpression	JAK/STAT inhibitors in combination with immune checkpoint inhibitors: Ruxolitinib + anti-PD-1 mAbs (NCT03681561) Combinatorial immune checkpoint blockade: Ipilimumab + Nivolumab (NCT02408861, NCT02304458) Anti-LAG3 mAb (MK-4280) + anti-PD-1 mAb (NCT03598608) Brentuximab + Nivolumab +/- Ipilimumab (NCT01896999)

CTL anergy through PD-1-PD-L1/2 interaction (HRS / TAM).	Adoptive cell therapy: Chimeric Antigen Receptor (CAR) CD30-targeting T-cells (NCT01316146, NCT01192464, NCT02690545, NCT02917083, NCT02259556, NCT03602157, NCT03049449) Bi-specific chimeric antibody constructs: INBRX-105 (PD-L1-CD137) provides a combination of PD-L1 blockade with concomitant T-cell co-stimulation through CD137 (4-1BB) agonism (NCT03809624)

NK cell inhibition mediated by TGF-ß and NKG2D-L interaction (HRS / MSC).	Bi-specific chimeric antibody constructs: AFM13 (CD30-CD16A) recruits NK cells via binding to CD16A as immune effector cells (NCT02321592) AFM13 + anti-PD-1 mAbs (NCT02665650)

CTL inhibition through TGF-ß, IL-10, Gal-1, TIMP1 and PGE2. Stimulation of CD4 T-cells differentiation towards Treg and Th2 phenotype through TGF-ß, IL-10, Gal-1, TIMP1 and PGE2. Chemo attraction of Treg and Th2 through CCL5 (fibroblasts), CCL17 and CC22 (HRS).	Immunomodulatory agents: Lenalidomide + anti-PD-1 mAbs (NCT02875067, NCT03015896, NCT01953692) Ibrutinib + anti-PD-1 mAbs (NCT02940301).

Th1 and CTL enhanced apoptosis through Fas ligand surface expression (HRS).	Induction of immunogenic cell death (ICD) of tumor cells with chemotherapy in combination with immune checkpoint inhibitors: Bendamustine + anti-PD-1 mAbs (NCT03343652) Bendamustine + Gemcitabine + anti-PD-1 mAbs (NCT03739619)

∗It should be noted that the trials involving inhibitors of deacetylase (HDACi) in cHL revealed a limited efficacy with significant hematological and electrolytic toxicities, rendering their future development difficult in the absence of predictive biomarkers [89, 90].

**Table 2:** Clinical trials of immune checkpoint inhibitors in cHL.

Study	Year	Drug, dose	Design	Phase	Clinical setting	No. of patients	ORR, %	CR, %	PFS
Ansell et al. (Checkmate 039) [7]	2015	Nivolumab, 3 mg/kg iv every 2 weeks until complete response, tumor progression, or excessive toxic effects	P	I	R/R	23	87	17	86% at 24 weeks

Armand et al. Checkmate 205 updated results (ASH 2018) [58]	2018	Nivolumab, 3 mg/kg iv every 2 weeks until disease progression, death, unacceptable toxicity, withdrawal of consent, or study end.∗ Patients in cohort C discontinue nivolumab after 1 year in persistent CR and could resume treatment if they relapsed within 2 years of the last dose	P Composed of 3 cohorts	II	R/R	243	69	16	Median PFS 15 months
			Cohort A (no exposition to BV)			63	65	29	Median PFS 17 months
			Cohort B (treatment with BV after auto-HSCT)			80	68	13	Median PFS 12 months
			Cohort C (treatment with BV before and/or after auto-HSCT failure)			100	73	12	Median PFS 15 months

Maruyama et al. [60]	2017	Nivolumab, 3 mg/kg iv on Day 1 each 14-day cycle.	P	II	R/R	17	81.3	23.5	60% at 6 months

Armand et al. (Keynote 013) [61]	2016	Pembrolizumab iv at a dose of 10 mg/kg every 2 weeks	P	I	R/R	31	65	16	69% at 24 weeks, 46% at 52 weeks

Zinzani et al. Keynote 087 updated results (ASH 2018) [63]	2018	Pembrolizumab, 200 mg iv every 3 weeks without premedication for a maximum of 24 months or until documented confirmed disease progression, intolerable toxicity, or investigator decision	P Composed of 3 cohorts	II	R/R	210	71.9	27.6	Median PFS 13.7 months
			Cohort 1 (progression after auto-HSCT and BV)			69	76.8	26.1	Median PFS 16.4 months
			Cohort 2 (progression after salvage chemotherapy and BV, but ineligible for auto-HSCT because of chemoresistant disease)			81	66.7	25.9	Median PFS 11.1 months
			Cohort 3 (progression after auto-HSCT, without BV)			60	73.3	31.7	Median PFS 19.4 months

Shi et al. (ORIENT-1) [68]	2019	Sintilimab, 200mg iv once every 3 weeks, until disease progression, death, unacceptable toxicity or withdrawal of consent, for a maximum of 24 months	P	II	R/R	92	80,4	34	77.6% at 6 months

Herbaux et al. [71]	2017	Nivolumab, 3 mg/kg iv once every 2 weeks without premedication until disease progression or unacceptable toxicity as assessed by investigators	R	-	R/R after allo-HSCT	20	95	42	58.2% at 12 months

Haverkos et al. [72]	2017	Nivolumab 3mg/kg iv every 2 weeks (n=28) or Pembrolizumab 200mg iv every 3 weeks (n=2)	R	-	R/R after allo-HSCT	30	79	50	Median PFS 591 days

BV = brentuximab vedotin, CR= complete response, HSCT= hematopoietic stem cell transplantation, IV = intravenously, NA = Not Available, ORR= overall response rate, P= prospective, PFS= progression free survival, R= retrospective, R/R= relapsed or refractory disease.

∗Amendment in July 2014: patients continued treatment beyond progression if protocol-predefined criteria were met (i.e. stable performance status and deriving perceived clinical benefit). Patients treated beyond initial progression were required to discontinue in the event of further progression (>10% further increase in tumor burden).

**Table 3:** Adverse Events linked to immune checkpoint inhibitors in cHL.

Agent	Reference / Phase	No. of patients	Most Common AE	Grade 3 or 4 AEs	Comments
Nivolumab	Ansell et al. (Checkmate 039) [7]	23	*78*%* Drug-related adverse events of any grade; the most common (>10*%): (i) Rash (22%) (ii) Thrombocytopenia (17%) (iii) Pyrexia (13%) (iv) Fatigue (13%) (v) Diarrhea (13%) (vi) Nausea (13%) (vii) Pruritus (13%)	*22*%* Drug-related adverse events of grade 3*: (i) Myelodysplastic syndrome (ii) Pancreatitis (iii) Pneumonitis (iv) Stomatitis (v) Colitis (vi) Gastrointestinal inflammation (vii) Thrombocytopenia (viii) Increased lipase levels (ix) Decrease lymphocyte level (x) Leukopenia	(i) Two patients discontinued treatment because of drug toxicity. (ii) AE reversible in all the patients except the 2 who discontinued.
	Younes and al. (Checkmate 205) [8]	80	*89*%* Drug-related adverse events of any grade; the most common (>10*%): (i) Fatigue (25%) (ii) IRR (20%) (iii) Rash (15%) (iv) Arthralgie (14%) (v) Pyrexia (14%) (vi) Nausea (13%) (vii) Diarrhea (10%) (viii) Pruritus (10%)	*21*%* Drug related grade 3*: (i) Neutropenia (5%) (ii) Increased lipase levels (3%) (iii) Increased ALT (3%) (iv) Increased AST (3%) (v) Abdominal pain (3%) (vi) Dyspnea (1%) (vii) Pneumonia (1%) (viii) Hepatitis (1%) (ix) Rash (1%) (x) Arthritis (1%) (xi) Syncope (1%) *4*%* Drug-related AE grade 4*: (i) Increased lipase (3%) (ii) Decreased neutrophil count (1%)	AE leading to discontinuation: (i) Treatment related autoimmune hepatitis (1 patient) (ii) Treatment related increased ALT and ASAT concentrations (1 patient) (iii) Death from multi-organ failure (1 patient) not treatment-related.
	Armand et al. (Checkmate 205) [57]	243	*Drug-related AE any grade*: (i) Fatigue (23%) (ii) Diarrhea (15%) (iii) Infusion-related reaction (14%) (iv) Rash (12%) (v) Pruritus (10%) (vi) Nausea (10%) *Immune-mediated AE*: (i) Hypothyroidism /thyroiditis (12%) (ii) Rash (9%) (iii) Hepatitis (5%) (iv) Pneumonitis (4%) (v) Hyperthyroidism (2%) (vi) Diabetes mellitus <1%	*Drug-related AE grade 3-4*: (i) Lipase increased (5%) (ii) Neutropenia (3%) (iii) ALT increased (3%) (iv) AST increased (2%)	7% discontinue treatment because of drug-related AE: (i) Pneumonitis (2%) (ii) Autoimmune hepatitis (1%)
	Maruyama and al. [60]	17	*Most common adverse events* (i) Pyrexia (41.2%) (ii) Pruritus (35.3%) (iii) Rash (35.3%) (iv) Hypothyroidism (29.4%)	*23.5*%* grade 3 or 4 AEs*: (i) Anemia (ii) Lymphopenia (iii) Thrombocytopenia (iv) Pyrexia (v) Hepatic function abnormal (vi) Pneumonia (vii) Hyponatremia (viii) Fulminant type 1 diabetes mellitus (ix) Interstitial lung disease (x) Rash	6 serious AE in 3 patients (all judged drug-related): (i) Pyrexia (ii) Hepatic function abnormal (iii) Hyponatremia (iv) Fulminant type 1 diabetes mellitus∗ (v) Interstitial lung disease∗ (vi) Rash ∗ Led to treatment discontinuation

Pembrolizumab	Armand et al. (Keynote 013) [61]	31	*68*%* Drug-related AE* (i) Hypothyroidism (16%) (ii) Nausea (13%) (iii) Diarrhea (16%) (iv) Pneumonitis (10%)	*16*%* Drug-related grade 3 AEs*: (i) Colitis (ii) Increased ALT and AST levels (iii) Nephrotic syndrome (iv) Joint swelling (v) Back pain (vi) Axillary pain	(i) Two patients discontinued due to grade 2 pneumonitis and grade 3 nephrotic syndrome
	Chen and al. (Keynote 087) [62]	210	*TRAE grade 1 or 2*: (i) Hypothyroidism (11.9%) (ii) Pyrexia (10%) (iii) Fatigue (8.6%) (iv) Rash (7.6%) (v) Diarrhea (6.2%)	*6.4*%* TRAE grade 3/4*: (i) Neutropenia (2.4%) (ii) Dyspnea (1%) (iii) Diarrhea (1%) (iv) Pyrexia (0.5%) (v) Cough (0.5%) (vi) Fatigue (0.5%) (vii) Hypothroidism (0.5%)	(i) 4.3% discontinued because of TRAEs (ii) 12.4% experienced TRAEs resulting in treatment interruptions.
	Zinzani et al. Keynote 087 updated results (ASH 2018) [63]	210	*72.9*%* TRAE of any grade*: (i) Hypothyroidism (14.3%) (ii) Pyrexia (11.4%) (iii) Fatigue (11%) (iv) Rash (11%)	*11.9*%* TRAE grade 3/4*: (i) Neutropenia (2.4%) (ii) Diarrhea (1.4%)	6.7% of patients discontinued treatment due to TRAE

Avelumab	Chen et al. [91]	31	*Treatment-related AE of any grade*: (i) IRR (26.7%) (ii) Nausea (20%) (iii) Rash (20%) (iv) Fatigue (13.3%)	*36.7*%* grade ≥3 AEs* – details NA	2 patients (6.7%) discontinued treatment due to IRR

Sintilimab	Shi et al. (ORIENT-1) [68]	96	*93*%* treatment-related AE of any grade*: (i) Pyrexia 41% (ii) Hypothyroidism 20% (iii) Increased ALT 14% (iv) Pneumonitis 11% (v) Infusion reaction 9% (vi) Rash 11% (vii) Increase AST 8% (viii) Decreased platelet counts 10% *54*%* immune-related AE*	*18*%* treatment-related AE grade 3 or 4* *11*%* Drug-related serious adverse events*: (i) Pneumonitis 3% (ii) Lung infection 3% (iii) Infusion reaction 2% (iv) Upper respiratory tract infection 1% (v) Liver function abnormality 1% (vi) Decreased platelet counts 1% (vii) Peripheral neuropathy 1% (viii) Hyperthyroidism 1%	3% (3 patients) discontinued treatment due to adverse events (i) 1 patient with grade 2 pneumonitis (ii) 1 patient with grade 4 liver function abnormality (iii) 1 patient with grade 3 pneumonitis and grade 4 decreased platelet count.

IRR = infusion related reactions; AE = adverse event; TRAE = treatment-related AE; NA = not available.
